# Climate and Vector Borne Pathogens: Challenges of the Present and of the Future

**DOI:** 10.1155/2019/4298716

**Published:** 2019-10-01

**Authors:** Dimosthenis Chochlakis, Snežana Tomanović, Emmanouil Angelakis

**Affiliations:** ^1^Laboratory of Clinical Microbiology and Microbial Pathogenesis, Unit of Zoonoses, School of Medicine, University of Crete, Heraklion, Greece; ^2^Department for Medical Entomology, Institute for Medical Research, University of Belgrade, Belgrade, Serbia; ^3^Aix-Marseille University, Institut de Recherche pour le Développement (IRD), Assistance Publique Hôpitaux de Marseille (APHM), Microbes, Evolution, Phylogénie et Infections, IHU (Institut Hospitalo-Universitaire)-Méditerranée Infection, Marseille, France

It is thought that 75% of emerging diseases are related to zoonotic diseases; therefore, any change in the ecology that can affect wildlife can also affect human health [1]. The transmission of infectious diseases should be considered through an ecological context. As regards those which are indirectly spread through a vector (mosquito, tick, flea, etc.), they and their respective vectors depend, to a large extent, on local microclimates, the so-called climatic shell (temperature, rainfall, rising sea levels, wind, sunshine, etc.) [2]. Climate changes may affect the vector itself and its ability to come into contact with humans or may lead to a prolongation of the transmission period. Vectors can adapt to changes in climate by altering their geographical distribution or by undergoing an evolutionary “response” to external changes. Furthermore, human activity (migrations, vector control practices (deparasitization, spraying, etc.), deforestation, land use change, the development of intensive crops, water storage, and the expansion of cities in suburban areas) may also affect transmission.

To understand the linkage among multiple factors and predict, to a certain extent, the distribution of vector-borne pathogens, several models are currently used. Experimental models contain a multitude of meteorological data and have been developed to describe the “bioclimatic envelope” for various vector-borne pathogens. Moreover, there is an ever-growing dynamic of the combination of climatic models with the field study which uses spatial analysis methods with the ultimate goal of studying various meteorological and environmental factors. Data are retrieved either from land or remote sensors or from satellites (GIS) and used for the analysis of the complex pathways involved[3].

Further to the above-mentioned, mathematical models and neural networks are now used to understand various parameters/factors/events in several areas of everyday life such as economy, environment, behaviors, migration (of humans and of animals), diseases (infectious or not), etc [4]. The goal is to move from ex post recording of events to their ex ante view, by processing existing and past data in order to make, as much as possible, the forecasting of future events (whether long-term, either short-term) feasible [5]. In vector-borne diseases, the information gathered daily is enormous and the need to exploit it in order to study not only their current behavior (spreading, distribution to the population of humans and animals, etc.) but also their future development. To do so, the need to introduce different approaches such as climatic and mathematical models, classical statistics, and Artificial Neural Networks seems to be unavoidable.

The purpose of current special issue was to put one more stone in the understanding of the link between climate and vector-borne diseases, as well as, to the tools that should be used to achieve this goal. The issue contains collection of five papers covering different aspects that one way or another, directly or indirectly cause a turn in the distribution of vector-borne diseases.

The paper “Application of kDNA Minicircle PCR-RFLP to Characterize *Leishmania donovani* Clinical Isolates Obtained from Post-Kala-Azar Dermal Leishmaniasis in Eastern Nepal” by O. Pokherel and coauthors focuses on molecular-epidemiology approach as a tool in revealing the transmission dynamics of visceral leishmaniasis (VL), vector-borne diseases transmitted by sandflies. Post-kala-azar dermal leishmaniasis (PKDL) is a skin manifestation of VL, and both forms of the disease are still considered as a major public health problem in South Asian countries such as Nepal. Using highly efficient molecular assays, the authors performed genotyping of VL and PKDL parasite isolates from Nepal. Both methods, with different resolution, showed separation of isolates in two groups. One group comprises isolates originating from hill districts and VL patients, while isolates clustered to the other group originate from low-land districts and both VL and PKDL patients. Based on these results, the authors propose the need for further study with large number of samples for systematic characterization of the clinical isolates to track the molecular-epidemiology of the *L. donovani* causing VL and the role of PKDL as a reservoir.

One of the emerging pathogens, widely distributed in aquatic environment, is *Shewanella algae*. Bacteremia is a major manifestation of *S. algae* infections, and there are increasing reports of antibiotic-resistant strains. However, little is known about the genomic characteristics of human bacteremic *S. algae*. In the paper “Genome Sequence of Colistin-Resistant Bacteremic *Shewanella algae* Carrying the Beta-Lactamase Gene bla_OXA-55_,” Y.-J. Chen and coauthors determined the whole genome sequence of a colistin-resistant *S. algae* strain isolated from blood. The authors proposed continuous surveillance for the emergence of *S. algae* and noted that the widespread environmental nature of the pathogen raises the concern for its role as a resistance reservoir.

Mosquitoes are the most important vectors of diseases on the global scale. The paper “Seasonal and Gender Differences in Presence of *Rickettsia felis* and Blood Meals Provide Additional Evidence of a Vector Role for Mosquitoes” by J. Zhang and coauthors indicates possible role of these vectors in transmission of rickettsial species vectored predominantly by fleas. While recent evidence has suggested mosquitoes could be infected with *R. felis*, there is little information about the exact role. For the purpose of this study, mosquitoes were collected monthly between 2013 and 2014 from the same residential dwelling at Yangzhou, China. Prevalence of *Rickettsia* and blood meal in mosquitoes were detected by PCR. Observed seasonal and gender differences of *R. felis* and blood-meal presence suggest the possible transstadial and transovarial transmission of the pathogen. The authors point to the need for further research of *R. felis* infection model within the full life cycle of mosquitoes to unambiguously prove the potential vector role of mosquitoes in transmission of this organism.

Moving on to the yellow fever virus, it has been responsible for thousands of deaths in the last decades. The article entitled “The Importance of Coordinated Actions in Preventing the Spread of Yellow Fever to Human Populations: The Experience from the 2016-2017 Yellow Fever Outbreak in the Northeastern Region of São Paulo State” by M. J. L. Siconelli and coauthors reports the features of the recent yellow fever (YF) outbreak in nonhuman primates (NHPs) in Brazil, the molecular characterization of a YF virus isolated during this period, and the importance of these findings to initiate coordinated measures among all public health services to prevent the occurrence of human cases. Yellow fever is a zoonotic arthropod-borne viral disease characterized by a sylvatic and urban cycle. The YF virus sylvatic cycle occurred in the peri-urban areas of the northeastern region of São Paulo state, but no human cases were reported during this period, showing that integrated actions between human, animal, and environmental health professionals were critical to restrain the virus to the sylvatic cycle.

We, as the guest editors, would like to thank all the authors of this special issue for contributing the high-quality papers. We would also like to thank the referees who have critically evaluated the papers and significantly influenced the quality standards of our special issue. Finally, we hope that the readers will find this special issue useful and informative.
